# Screening for Resistant Bacteria, Antimicrobial Resistance Genes, Sexually Transmitted Infections and *Schistosoma* spp. in Tissue Samples from Predominantly Vaginally Delivered Placentae in Ivory Coast and Ghana

**DOI:** 10.3390/pathogens12080999

**Published:** 2023-07-30

**Authors:** Roman Franz, Andreas Hahn, Ralf Matthias Hagen, Holger Rohde, Kirsten Alexandra Eberhardt, Stephan Ehrhardt, Jana Baum, Lisa Claussen, Torsten Feldt, Rebecca Hinz, Dana Barthel, Carola Bindt, Harry Tagbor, Samuel Blay Nguah, Mathurin Koffi, Thomas Köller, Philipp Warnke, Frederik Pankok, Stefan Taudien, Hagen Frickmann, Stefanie Schoppen

**Affiliations:** 1 Department of Microbiology and Hospital Hygiene, Bundeswehr Hospital Hamburg, 20359 Hamburg, Germany; romanfranz@bundeswehr.org; 2 Department of Microbiology and Hospital Hygiene, Bundeswehr Hospital Berlin, 10115 Berlin, Germany; 3 Institute for Medical Microbiology, Virology and Hygiene, University Medicine Rostock, 18057 Rostock, Germany; andreas.hahn@uni-rostock.de (A.H.); thomas.koeller@med.uni-rostock.de (T.K.); philipp.warnke@med.uni-rostock.de (P.W.); 4 Department of Microbiology and Hospital Hygiene, Bundeswehr Central Hospital Koblenz, 56070 Koblenz, Germany; ralfmatthiashagen@bundeswehr.org; 5 Institute of Medical Microbiology, Virology and Hygiene, University Medical Center Hamburg-Eppendorf (UKE), 20251 Hamburg, Germany; rohde@uke.de; 6 Department of Tropical Medicine, Bernhard Nocht Institute for Tropical Medicine & I. Department of Medicine, University Medical Center, 20359 Hamburg, Germany; k.eberhardt@bnitm.de; 7 Division of Hygiene and Infectious Diseases, Institute of Hygiene and Environment, 20539 Hamburg, Germany; 8 Department of Epidemiology, Johns Hopkins Bloomberg School of Public Health, Baltimore, MA 21205, USA; sehrhar6@jhu.edu; 9 Clinical Research Unit, Bernhard Nocht Institute for Tropical Medicine Hamburg, 20359 Hamburg, Germany; jana-baum@hotmail.com; 10 Department of Anaesthesiology and Intensive Care, Asklepios Klinik Altona, 22763 Hamburg, Germany; l.claussen@asklepios.com; 11 Department of Gastroenterology, Hepatology and Infectious Diseases, Medical Faculty and University Hospital Düsseldorf, Heinrich Heine University Düsseldorf, 40225 Düsseldorf, Germany; torsten.feldt@med.uni-duesseldorf.de; 12 Department of Clinical Microbiology, Synlab MVZ Hamburg GmbH, 22083 Hamburg, Germany; rebecca.hinz@synlab.com; 13 Department of Child and Adolescent Psychiatry, Psychotherapy, and Psychosomatics, University Medical Center, 20251 Hamburg, Germany; d.barthel@uke.de (D.B.); bindt@uke.de (C.B.); 14 School of Medicine, Department of Community Health, University of Health and Allied Sciences, Ho PMB 31, Ghana; htagbor@uhas.edu.gh; 15 School of Medicine and Dentistry, Kwame Nkrumah University of Science and Technology, Kumasi AK-385-1973, Ghana; sbnguah@gmail.com; 16 Université Jean Lorougnon GUEDE, UFR Environnement-Santé, Laboratoire des Interactions Hôte-Microorganismes-Environnement et Evolution (LIHME), Daloa BP 150, Côte d’Ivoire; m9koffi@yahoo.fr; 17 Institute for Infection Control and Infectious Diseases, University Medical Center Göttingen, 37075 Göttingen, Germany; frederik.pankok@med.uni-goettingen.de (F.P.); stefan.taudien@med.uni-goettingen.de (S.T.); 18 Department of Health and Social Science, Hochschule Fresenius, 20148 Hamburg, Germany

**Keywords:** placenta, antimicrobial resistance, sexually transmitted infections, schistosomiasis, epidemiology, pregnancy, Ghana

## Abstract

Medical complications during pregnancy have been frequently reported from Western Africa with a particular importance of infectious complications. Placental tissue can either become the target of infectious agents itself, such as, e.g., in the case of urogenital schistosomiasis, or be subjected to contamination with colonizing or infection-associated microorganisms of the cervix or the vagina during vaginal delivery. In the retrospective cross-sectional assessment presented here, the quantitative dimension of infection or colonization with selected resistant or pathogenic bacteria and parasites was regionally assessed. To do so, 274 collected placental tissues from Ivory Coastal and Ghanaian women were subjected to selective growth of resistant bacteria, as well as to molecular screening for beta-lactamase genes, *Schistosoma* spp. and selected bacterial causative agents of sexually transmitted infections (STI). Panton–Valentine-negative methicillin-resistant *Staphylococcus aureus* (MRSA) was grown from 1.8% of the tissue samples, comprising the *spa* types t008 and t688, as well as the newly detected ones, t12101 (n = 2) and t12102. While the culture-based recovery of resistant Enterobacterales and nonfermentative rod-shaped Gram-negative bacteria failed, molecular assessments confirmed beta-lactamase genes in 31.0% of the samples with multiple detections of up to four resistance genes per sample and *bla*_CTX-M_, *bla*_IMP_, *bla*_GES_, *bla*_VIM_, *bla*_OXA-58_-like, *bla*_NDM_, *bla*_OXA-23_-like, *bla*_OXA-48_-like and *bla*_KPC_ occurring in descending order of frequency. The beta-lactamase genes *bla*_OXA-40/24_-like, *bla*_NMC_A/IMI_, *bla*_BIC_, *bla*_SME_, *bla*_GIM_ and *bla*_DIM_ were not detected. DNA of the urogenital schistosomiasis-associated *Schistosoma haematobium* complex was recorded in 18.6% of the samples, but only a single positive signal for *S. mansoni* with a high cycle-threshold value in real-time PCR was found. Of note, higher rates of schistosomiasis were observed in Ghana (54.9% vs. 10.3% in Ivory Coast) and Cesarean section was much more frequent in schistosomiasis patients (61.9% vs. 14.8% in women without *Schistosoma* spp. DNA in the placenta). Nucleic acid sequences of nonlymphogranuloma-venereum-associated *Chlamydia trachomatis* and of *Neisseria gonorrhoeae* were recorded in 1.1% and 1.9% of the samples, respectively, while molecular attempts to diagnose *Treponema pallidum* and *Mycoplasma genitalium* did not lead to positive results. Molecular detection of *Schistosoma* spp. or STI-associated pathogens was only exceptionally associated with multiple resistance gene detections in the same sample, suggesting epidemiological distinctness. In conclusion, the assessment confirmed considerable prevalence of urogenital schistosomiasis and resistant bacterial colonization, as well as a regionally expected abundance of STI-associated pathogens. Continuous screening offers seem advisable to minimize the risks for the pregnant women and their newborns.

## 1. Introduction

Pregnant women are a particularly vulnerable population in the case of exposure to infectious disease agents in resource-limited tropical settings like Western Africa, as is particularly well documented for Ghana. In two independent studies, maternal mortality rates of about 1% have been calculated for Ghanaian mothers [1,2]. Infections arising from the genital tract, as well as from other sites, with and without sickle cell disease and hypertensive disease have been shown to account for relevant proportions of this death toll [1,2], with puerperal sepsis accounting for 8.9% of deaths [1]. Fetal deaths in Ghana, in contrast, were found to be associated with fetal infections in 9.7% to 13.0% of cases [3,4] and placental inflammation in up to 24.8% of cases [4]. As repeatedly demonstrated, viral, bacterial, mycobacterial and parasitological infections threaten the health of mother and fetus [5,6,7,8,9,10,11,12,13,14,15,16]. For Ivory Coast, fewer respective studies have been published, but available data nevertheless indicate relevant pregnancy-associated infection risks [17].

The placenta plays a role as an interface between maternal and fetal organisms [18,19,20,21,22,23], making it a tissue of interest as diagnostic material for the screening for pregnancy-associated infections. In the study presented here, vaginally delivered placental tissue from Ivory Coastal and Ghanian mothers was analyzed for a number of less frequently assessed pathogens to shed light on the local epidemiological situation. *Schistosoma haematobium*, the causative agent of female genital schistosomiasis, is known to be prevalent in Ghana with regionally varying prevalence rates [24,25,26,27]. The same applies to Ivory Coast, where *S. haematobium* × *S. bovis* hybrids have been described next to *S. haematobium* and *S. mansoni* [28,29,30,31,32,33,34,35,36]. In pregnant Ghanaian women, the disease is known to be associated with anemia [37]. Multidrug-resistant bacteria are common in Ghanian and Ivory Coastal patients as well [38,39,40,41,42]. While, in particular, resistant Gram-negative bacteria are frequent in urinary tract infections and bloodstream infections in Ghana [38,39], their proportion in neonatal and pregnancy-associated infections seems to be still low [43,44]. In small cross-sectional studies from Ivory Coast, extended-spectrum beta-lactamase (ESBL)-expressing Enterobacterales and metallo-beta-lactamase-positive *Pseudomonas aeruginosa* have been described [41,42]. Increased antimicrobial resistance rates are also quite common in bacterial agents, causing sexually transmitted infections (STI) like gonococci in Ghana [45]. STI and sexual HIV transmission are still frequent in Ghanaian individuals, although HIV seropositivity rates have declined in Ghanaian female sex workers over the last decades because of the implementation of prevention programs [46], and sociological assessments did not reveal a particularly increased STI risk for pregnant Ghanaian women because of higher frequencies of sexual contacts of their partners outside the main relationship [47]. In Ivory Coast, increased STI rates have been reported predominantly for sex workers [48,49,50,51,52,53]; however, scarcely available data on non-preselected populations suggest low to intermediate one-digit percentages for gonococci and chlamydia as well [54].

In summary, the study was conducted (a) to contribute to the epidemiological information on Ivory Coastal and Ghanaian female genital schistosomiasis affecting placental tissue, as well as (b) to find traces of multidrug-resistant vaginal bacterial colonization and of vaginal STIs on placenta tissue caused by contamination events during vaginal delivery. For this purpose, placental tissue samples from pregnant Ivory Coastal and Ghanaian women were subjected to culture-based and molecular screening for the microbial targets.

## 2. Materials and Methods

### 2.1. Ethics

All procedures were conducted in accordance with the Helsinki Declaration. The Child Development Study (CDS) was approved by the responsible ethical committees in each country, namely the national ethical committee in Cote d’Ivoire (Ref: 4169/MHSP), the ethical committee of the Kwame Nkrumah University of Science and Technology in Kumasi, Ghana (Ref: CHRPE/KNUST/KATH/01_06_08) and the ethical committee of the chamber of physicians in Hamburg, Germany (Ref: PV3020). All women participating in the CDS have provided written informed consent.

### 2.2. Placenta Tissue Sample Collection and Storage

A total of 274 tissue samples from vaginally delivered placentae of Ivory Coastal and Ghanaian mothers were collected in the course of the CDS, which had been conducted to assess the impact of communicable and noncommunicable disease on infant development in the Western African tropics [55,56] at the study sites Komfo Anokye Teaching Hospital in Kumasi (Ghana) and Abobo Community Hospital in Abidjan (Ivory Coast). Available epidemiological data are provided in Table 1 below. As indicated in Table 1 by varying denominators, not all epidemiological information was available for each sample. Incomplete epidemiological data were not used as criteria for the exclusion of samples from the assessment. Samples were included in the assessment if at least twice 200 mg tissue was available. The samples were stored at −80 °C and split in two halves prior to further assessment in the course of this retrospective cross-sectional study.

### 2.3. Culture-Based Assessments

One-half (200 mg) of each sample volume was incubated in nonselective Mueller–Hinton (Becton-Dickinson, Heidelberg, Germany) broth at 36 ± 1 °C for 24 h as an enrichment step after deep-frozen sample storage. Afterward, the incubated broth was subcultured on selective agars like Brilliance ESBL agar (Oxoid, Basingstoke, UK) for 3rd-generation cephalosporine-resistant Enterobacterales, CHROMagar *Acinetobacter* (Mast Diagnostika, Reinfeld, Germany) for *Acinetobacter* spp. and chromID VRE agar (bioMeriéux, Nürtingen, Germany) for vancomycin-resistant *Enterococcus* spp. (VRE), as well as CHROMagar MRSA (Mast Diagnostika, Reinfeld, Germany) for methicillin-resistant *Staphylococcus aureus* (MRSA) at 36 ± 1 °C for 24 h. Suspicious colonies in line with the manufacturers’ recommendations were subjected to differentiation using a Shimadzu/Kratos “AXIMA Assurance” MALDI TOF MS device (Shimadzu Deutschland GmbH, Duisburg, Germany) and the “IVD-mode VitekMS-ID” database version 3.2.0.-6 (bioMérieux, Marcy-l′Étoile, France), next to resistance testing applying AST-P654 and AST-N429 cards in a VITEK-II automated device (bioMérieux) with interpretation according to the EUCAST (European Committee on Antimicrobial Resistance Testing) standard (clinical breakpoint version 13).

Of note, DNA of Gram-negative rod-shaped bacteria obtained from the respective selective agars was released by three-times-repeated freeze–thawing and subjected to the beta-lactamase-specific real-time PCRs as described below. DNA of MRSA isolates was subjected to Panton–Valentine leucocidin gene-specific PCR and to spa typing as previously described [57,58].

### 2.4. Nucleic Acid Extraction and Amplification from Primary Sample Materials

The nucleic acids from the other half of each deep-frozen placenta sample that was subjected to molecular assessments were extracted applying the EZ1&2 DNA tissue kit protocol on EZ1 automates (Qiagen, Hilden, Germany) according to the manufacturer’s instructions after bead-beating-based tissue lysis. The bead-beating procedure was performed as follows: 200 mg tissue volumes together with n = 3.5 mm sized steel beads were subjected to liquid-nitrogen freezing of the tubes and subsequent tissue lysis for 5 min at 30/s applying a TissueLyser LT device (Qiagen, Hilden, Germany). Obtained nucleic acids were measured using a Pico 100 Picodrop microliter spectrophotometer (Picodrop Ltd., Hinxton, UK) according to the manufacturer’s instructions. The mean value ± standard deviation (SD) of the measured DNA concentrations was 237.7 ng/µL ± 84.4 ng/µL. Eluates were deep frozen at −80 °C prior to the PCR assessments.

All samples were subjected to previously published real-time PCR assays targeting beta-lactamase genes, bacterial agents causing sexually transmitted infections (STI) and *Schistosoma* spp. prevalent in Ghana and Ivory Coast, i.e., *Schistosoma haematobium* complex, as well as *Schistosoma mansoni* complex on RotorGene Q cyclers (Qiagen, Hilden, Germany). The performed beta-lactamase PCR targets comprised the genes *bla*_CTX-M_, *bla*_VIM_, *bla*_IMP_, *bla*_NDM_ and *bla*_KPC_ as described by van der Zee and colleagues [59], *bla*_OXA-23_-like, *bla*_OXA-40/24_-like, *bla*_OXA-48_-like and *bla*_OXA-58_-like as described by Probst and colleagues [60] and *bla*_GES_, *bla*_NMC-A/IMI_, *bla*_BIC_ and *bla*_SME_ as described by Berneking and colleagues [61], as well as *bla*_GIM_ and *bla*_DIM_ as described by Poirel and colleagues [62] with added hybridization probes specifically designed for this study. The applied STI PCRs targeted the *polA* gene of *Treponema pallidum* [63], a *Chlamydia trachomatis* cryptic plasmid sequence for the *Chlamydia trachomatis* screening and the *pmpH* gene for the discrimination of the *Chlamydia trachomatis* serovars A–K from the serovars L1–L3 [64], a sequence of the MgPA operon of *Mycoplasma genitalium* [65,66] and the multi-copy *opa* genes, as well as the *porA* pseudogene of *Neisseria gonorrhoeae* [67]. In the latter case, positive results for both genomic targets were expected to confirm the diagnosis of an *N. gonorrhoeae* infection [67]. The *pmpH* gene-based serovar discrimination of *C. trachomatis* included the use of a pan-serovar-specific hybridization probe, as well as a serovar-A–K-specific probe, resulting in the diagnosis of an L1–L3 serovar if the pan-serovar-specific hybridization probe provided a positive signal and the serovar-A–K-specific probe did not [64]. Finally, the real-time PCRs targeting the multi-copy sequences *Sm1-7* of the *S. mansoni* complex and *Dra1* of the *S. haematobium* complex [68] were conducted. The oligonucleotides of the various target-specific real-time PCRs are shown in Table A1. The real-time PCR assays were conducted as described [59,60,61,62,63,64,65,66,67,68] with minor modifications. Details are provided in Table A2, Table A3 and Table A4. There were no specific cycle threshold (Ct) cut-offs within the amplification range. Instead, typical sigmoid-shaped amplification curves as assessed by experienced investigators were considered as likely specific, irrespective of the measured Ct values. Both qualitative and semiquantitative assessments of the real-time PCR results were performed. Quality-control procedures comprised the inclusion of a plasmid-based positive control (sequence inserts in a pEX A128 vector backbone (eurofins Genomics, Luxembourg)) or a positive control gblock (Integrated DNA Technologies, Coralville, ID, USA) (positive control sequences provided in Table A1) and a PCR-grade water-based negative control in each run. Detection thresholds for each PCR were calculated based on the 10-fold dilution steps of the positive control plasmids or gblocks, applying the internet-based software “Calculator for determining the number of copies of a template” (https://cels.uri.edu/gsc/cndna.html (accessed on 4 May 2023)). The calculated detection thresholds, ranging from <10^2^ copies/µL to 5 × 10^2^ copies/µL, are indicated in Table A1. A phocid herpes virus DNA-based real-time PCR, as described recently [69], was conducted as a combined extraction and inhibition control for each sample.

### 2.5. Statistical Assessment

Considering the low case numbers and the explorative character of the study, only descriptive assessments and simple statistical operations were performed. The applied software tools were Microsoft Excel from the Microsoft Office Package 2019 (Microsoft, Redmond, Washington, DC, USA) and GraphPad InStat, version 3.06, 32 bit for Windows (GraphPad Software Inc., San Diego, CA, USA). For a superficial assessment of potential associations with epidemiological data, nonparametric Mann–Whitney U testing was applied in the case of numerical parameters, Fisher’s exact test with two-sided *p*-values, the approximation of Woolf for the 95%-confidence interval calculation and Yate’s continuity correction in the case of dichotomous parameters, as well as the Chi-square test of independence in the case of nondichotomous, nominally scaled parameters.

## 3. Results

### 3.1. Growth of Resistant Bacteria on Selective Agars after Broth Enrichment

From the 274 placenta tissue samples, specific microbial growth was not observed on the selective agars for third-generation cephalosporin-resistant Enterobacterales, vancomycin-resistant enterococci and *Acinetobacter* spp. in the course of the assessment. Of note, growth of *Brucella intermedia* (formerly *Ochromobactrum intermedium*) was detected on the *Acinetobacter* spp.-selective agar in a single instance (0.4%, 1/274), as identified with MALDI-TOF-MS, and confirmed applying a previously published 16S rRNA gene-sequencing protocol with the forward primer 16S8_27 5′-AGAGTTTGATCMTGGCTCAG-3′ and the reverse primer 16S519 5′-GWATTACCGCGGCKGCTG-3′ [70]. In contrast, five MRSA isolates (1.8%, 5/274) could be grown on selective agar. The detected *spa* types comprised t008 (n = 1) and t688 (n = 1), as well as the newly assigned ones, t21101 (n = 2) and t21102 (n = 1). Of note, the *spa* types most closely related to t21101 are t017 and t9852 (one nucleotide mismatch each), and the *spa* type most closely related to t20102 is t7443 (eight mismatching nucleotides). The Panton–Valentine leucocidin gene was detected in none of the MRSA isolates. Focusing on antimicrobial resistance beyond the beta-lactam antibiotics in the MRSA isolates, tetracycline resistance was observed in four out of five isolates (t008, t688 and both t21101 isolates) and clindamycin resistance in two out of five isolates (t688 and one out of two t21101 isolates), while only the t008 isolate showed resistance against rifampicin and the t688 isolate was resistant against erythromycin. No acquired resistance was phenotypically recorded against gentamicin, cotrimoxazole, vancomycin, fosfomycin, fusidic acid, linezolid, daptomycin and tigecycline in the MRSA isolates.

### 3.2. Samples Included in the Molecular Assessments

**Figure 1:**
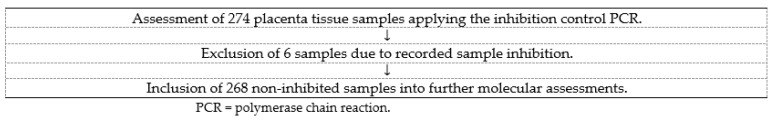
Flowchart visualizing the samples included in the molecular assessments.

The applied inhibition-control real-time PCR showed positive results in 268/274 (97.8%) of the nucleic acid extractions of the PhHV-(phocid herpes virus-)DNA-spiked samples. The mean value (±standard deviation SD) of the measured Ct values was 27.0 (±2.9). The remaining six samples with negative-inhibition-control PCR were excluded from any further molecular assessments. A flowchart showing the effect of this quality control procedure on the total sample count for the molecular assessments is visualized as Figure 1.

### 3.3. Molecular Detection of Genetic Resistance Determinants

Positive real-time PCR results for the assessed extended-spectrum beta-lactamase and carbapenemase genes were recorded in 83/268 (31.0%) noninhibited nucleic acid eluates of the placenta samples. The detected resistance genes comprised *bla*_CTX-M_ (n = 42), *bla*_IMP_ (n = 25), *bla*_GES_ (n = 20), *bla*_VIM_ (n = 12), *bla*_OXA-58_-like (n = 10), *bla*_NDM_ (n = 6), *bla*_OXA-23_-like (n = 5), *bla*_OXA-48_-like (n = 5) and *bla*_KPC_ (n = 2) in descending order of frequency. Details on the proportions of resistance-gene distribution, as well as on the recorded cycle threshold (Ct) values in real-time PCR, are shown in Table 2. A proportion of 38.6% (32/83) of samples with detected beta-lactamase genes showed real-time PCR positivity for more than a single resistance gene. In the 51 samples positive for only one screened resistance gene, the detected beta-lactamases comprised *bla*_CTX-M_ (n = 24), *bla*_GES_ (n = 14), *bla*_IMP_ (n = 7), *bla*_VIM_ (n = 3), *bla*_OXA-58_-like (n = 2) and *bla*_OXA-23_-like (n = 1). Double detections were seen in 22 instances, with *bla*_CTX-M_ and *bla*_OXA-48_-like (n = 3), *bla*_CTX-M_ and *bla*_IMP_ (n = 3), *bla*_VIM_ and *bla*_IMP_ (n = 3), *bla*_CTX-M_ and *bla*_VIM_ (n = 2), *bla*_NDM_ and *bla*_IMP_ (n = 2), *bla*_CTX-M_ and *bla*_GES_ (n = 2), *bla*_OXA-23_-like and *bla*_OXA-58_-like (n = 2), *bla*_VIM_ and *bla*_GES_ (n = 1), *bla*_IMP_ and *bla*_GES_ (n = 1), *bla*_NDM_ and *bla*_KPC_ (n = 1), *bla*_CTX-M_ and *bla*_OXA-58_-like (n = 1) and *bla*_IMP_ and *bla*_OXA-23_-like (n = 1) as the observed dual combinations. Triple detections of beta-lactamase genes were seen in eight samples with *bla*_CTX-M_ and *bla*_IMP_ and *bla*_OXA-58_-like (n = 3) as the most frequently recorded combination. The other detected triple combinations comprised *bla*_CTX-M_ and *bla*_IMP_ and *bla*_GES_ (n = 1), *bla*_CTX-M_ and *bla*_OXA-23_-like and *bla*_OXA-58_-like (n = 1), *bla*_VIM_ and *bla*_IMP_ and *bla*_OXA-48_-like (n = 1), *bla*_NDM_ and *bla*_VIM_ and *bla*_IMP_ (n = 1) and *bla*_NDM_ and *bla*_KPC_ and *bla*_IMP_ (n = 1). Finally, there were two cases with quadruple beta-lactamase detections. The observed combinations were *bla*_CTX-M_ and *bla*_IMP_ and *bla*_OXA-48_-like and *bla*_OXA-58_-like (n = 1), as well as *bla*_CTX-M_ and *bla*_NDM_ and *bla*_VIM_ and *bla*_GES_ (n = 1). The beta-lactamases *bla*_OXA-40/24_-like, *bla*_NMC_A/IMI_, *bla*_BIC_, *bla*_SME_, *bla*_GIM_ and *bla*_DIM_ were not detected within the assessed sample materials. Of note, the MRSA t008 isolate was grown from a sample also containing the beta-lactamase genes *bla*_CTX-M_ and *bla*_IMP_ and the MRSA t688 isolate from a sample also containing *blaCTX-M*, while no such associations were seen for the other MRSA spa types and for the *Brucella intermedia* isolate.

### 3.4. Molecular Detection of Sexually Transmitted Infections

From the 268 placenta samples included in the assessment, nonlymphogranuloma-venereum-associated *Chlamydia trachomatis* was recorded in three instances (3/268, 1.1%) and *Neisseria gonorrhoeae* in five instances (5/268, 1.9%). In two additional cases, suspicions of *N. gonorrhoeae* could not be confirmed because only one out of two PCRs was positive in each. DNA of *Treponema pallidum*, lymphogranuloma-venereum-associated *C. trachomatis* and *Mycoplasma genitalium* was not detected. Details including recorded cycle threshold (Ct) values of the real-time PCR assays are provided in Table 3. There were no coincidences of different sexually transmitted infections within the same individual observed. Of note, two chlamydial infections were recorded in individuals without parallel proof of beta-lactamases, while another identification of *C. trachomatis* succeeded in a sample with a concomitant detection of a *bla*GES resistance gene. From one of the two other *C. trachomatis*-positive samples, one of the MRSA t21101 isolates could be isolated. Two samples with confirmed gonococci and one sample with nonconfirmed gonococci (only positive in the *opa* gene PCR) were free of beta-lactamase detections, while beta-lactamase combinations were observed in three samples with confirmed gonococci (*bla*_CTX-M_ and *bla*_OXA-23_-like and *bla*_OXA-58_-like, *bla*_OXA-23_-like and *bla*_OXA-58_-like, as well as *bla*_NDM_ and *bla*_IMP_) and one sample with nonconfirmed gonococci (*bla*_OXA-23_-like and *bla*_OXA-58_-like).

### 3.5. Molecular Detection of Schistosoma Mansoni Complex and Schistosoma Haematobium Complex

A total of 50 positive real-time PCR signals for *Schistosoma haematobium* complex next to a single *S. mansoni* complex detection were observed. Thereby, one *S. haematobium* detection with a typical sigmoid-shaped amplification curve and a cycle threshold (Ct) value of 32 even occurred in a sample showing inhibition, resulting in a denominator of 269 for this single parameter. Details on the proportions of positive results and the recorded Ct values are provided in Table 4. From a total of 51/269 (19.0%) samples containing *Schistosoma* spp.-specific DNA, 37.3% (19/51) were also positive for other screened parameters. In particular for the 50 samples with *S. haematobium* complex DNA, n = 7 also contained *bla*_CTX-M_ genes, another n = 7 *bla*_GES_, n = 1 a combination of *bla*_CTX-M_ and *bla*_OXA-58_-like, n = 1 a combination of *bla*_CTX-M_ and *bla*_IMP_, n = 1 a combination of *bla*_GES_ and nonlymphogranuloma-venereum-associated *Chlamydia trachomatis*, n = 1 *bla*_VIM_ and n = 1 *bla*_OXA-58_-like. No concomitant detections were recorded for the *S. mansoni*-complex-DNA-containing sample.

### 3.6. Associations with Epidemiological Data as Observed in the Epidemiological Assessment

To exploratively associate the laboratory findings with available epidemiological information, a number of simplifications were introduced. To obtain sufficiently sized groups for the assessments, three clusters were formed comprising (a) cases with culturally grown resistant bacteria and/or molecular proofs of beta-lactamase genes, (b) cases with sexually transmitted infections and (c) cases with placental schistosomiasis. To avoid working with different denominators for culture-based diagnostic approaches and molecular diagnostic approaches, samples showing PCR inhibition were counted as negative for the respective PCR parameter as another simplification.

Applying these premises for the calculations, no associations were observed for any recorded epidemiological parameters and (a) cases with culturally grown resistant bacteria and/or molecular proofs of beta-lactamase genes, as well as (b) cases with sexually transmitted infections. For cases with placental schistosomiasis, a number of associations with a significance level *p* < 0.05 were found. First, a higher proportion of placental schistosomiasis was observed for Ghanaian women (54.9% (28/51)) compared to Ivory Costal women (10.3% (23/223)) (*p* < 0.0001). Second, women with placental schistosomiasis were more likely to deliver their offspring via Caesarian section (61.9% (13/21)) rather than via the vaginal route (14.8% (36/244)) (*p* < 0.0001). Third, the APGAR 1 score value of newborns from women with placental schistosomiasis (7.5 (±1.1)) was slightly lower than the APGAR 1 score value of newborns from women without this medical condition (7.9 (±1.1)) (*p* = 0.01). Fourth, placental schistosomiasis was more frequently observed in women possessing a freezer (33.3% (25/99)) compared to women without such a device (10.3% (18/175)) (*p* < 0.0001). Fifth, there was a hint of more placental schistosomiasis in women with better sanitary equipment, comprising 12.8% (19/148) schistosomiasis in women with a pit latrine compared to 24.7% (18/73) with an improved pit latrine and 26.4% (12/53) with a flush toilet (*p* = 0.03). Among the nonsignificant findings, it is remarkable that placentae delivered via Caesarian section showed a similar (*p* = 0.33) contamination rate with resistant bacteria and molecular resistance determinants compared to the placentae delivered via the vaginal route.

Details are provided in Table A5, Table A6 and Table A7.

## 4. Discussion

The study was conducted as an epidemiological assessment with vaginally delivered placenta tissue collected from Ivory Coastal and Ghanaian mothers. The basic assumption was that vaginally delivered placentae are similarly exposed to vaginal colonization flora like the newborn, and so, screenings for resistant bacteria and causative agents of sexually transmitted infections in placenta tissue might well reflect this exposure situation. As Ghana [24,25,26,27] and Ivory Coast [28,29,30,31,32,33,34,35,36] are known prevalence regions for schistosomiasis, the tissue was assessed for molecular hints of urogenital schistosomiasis as well.

The assessment led to various results. Although a considerable number of extended beta-lactamase (ESBL) and carbapenemase genes indicative for colonization with third-generation cephalosporin-resistant and carbapenem-resistant Gram-negative rod-shaped bacteria could be identified, culture-based isolation of such microorganisms was most likely prevented by unfavorable long-term storage and transport conditions of the samples, which is an undeniable limitation of the study. As an unexpected side finding, instead, *Brucella intermedia* (formerly *Ochrobactrum intermedium*) was isolated from *Acinetobacter*-selective agar. As infections with this low-pathogenic *Brucella* species are usually associated with immunocompromising medical conditions [71,72], the isolation was interpreted as indicative of a most likely harmless colonization event of the vagina. In contrast to the more vulnerable Gram-negative colonization flora, Gram-positive methicillin-resistant *Staphylococcus aureus* (MRSA) could still be grown from various samples. The identified *spa* type t008 is known to circulate in the Western African Nigeria [73,74], while the *spa* type t688 has been described from livestock, food products and patient samples from the Northern African Algeria and Egypt [75,76,77]. Next to previously known *spa* types, two newly observed ones were also found among the MRSA isolates. The new *spa* type t21101 is closely related to t017, a *spa* type of which a methicillin-susceptible variant is known to circulate in Nigeria [78], suggesting evolutionary selection of the now-observed Ghanaian lineage. Of note, *spa* type t21101 was isolated twice, and it remains uncertain whether this co-occurrence was due to regionally high abundance of this clone or due to direct transmission. The difference in clindamycin susceptibility between the two t21101 isolates might, however, speak against the latter option.

Focusing on the beta-lactamase genes detected by real-time PCR, the quantitative dominance of *bla*_CTX-M_ is not further surprising, as this genetic resistance determinant has been repeatedly described to be highly prevalent in Ghana [79,80,81,82,83,84,85,86,87,88,89,90,91,92,93] and also Ivory Coast [41]. In contrast, carbapenemases are still less frequently reported from Ghana and Ivory Coast with resistance genes like, e.g., *bla*_IMP_, *bla*_NDM_, *bla*_OXA-23_-like, *bla*_OXA-48_-like, including *bla*_OXA-181_, *bla*_OXA-58_-like and *bla*_VIM_ being associated with varying low prevalence rates or outbreaks [42,94,95,96,97,98,99,100,101,102,103,104,105]. The here-observed prevalence may contribute to the scarcely available epidemiological information regarding this topic. The recorded composition of detected carbapenemase genes matches the abovementioned previous reports for Ghana quite well with the addition of a low proportion of *bla*_KPC_ gene detections and a slightly higher quantity of *bla*_GES_ gene detections. The latter finding might be explained by the fact that *bla*_GES_ is rarely included in standard screening panels, although the occurrence of GES-type carbapenemases on the African continent has been repeatedly reported [106,107,108]. In the here-presented assessment, *bla*_GES_ was the second most frequently observed carbapenemase gene next to the primarily *Pseudomonas-aeruginosa*-associated carbapenemase-gene *bla*_IMP_, which was the most frequently observed one, and *bla*_VIM_ as the third most frequent carbapenemase gene. *Acinetobacter*-associated carbapenemase genes like *bla*_OXA-58_-like and *bla*_OXA-23_-like followed in the order of declining abundance, while carbapenemase genes typically found in Enterobacterales, like *bla*_NDM_, *bla*_OXA-48_-like and *bla*_KPC_, were only occasionally observed.

The distribution of the detected beta-lactamase genes over the sample collection seems noteworthy. First, there was no significant difference in the colonization rate of vaginally delivered placentae and placentae delivered via Caesarian section. This finding hints at secondary contamination events during or after surgery. Second, while a majority of samples was free of the assessed resistance genes, all molecular resistance-gene detections were focused on less than one-third of the samples. Within the subpopulation of those 83 resistance-gene-positive samples, nearly 40% were positive for more than one resistance gene and more than 10% for even more than two. As the respective clinical information was not available, it can only be speculated that selection pressure caused by the intake of antimicrobial drugs might have contributed to the observed clustering of beta-lactamase genes in a minority of screened individuals. As several resistance genes can be harbored by the same bacterium, the detection of multiple resistance genes does not necessarily mean evidence for colonization with multiple resistant bacteria; however, at least in some instances this is likely to have been the case. Because the contamination of placenta tissue during vaginal delivery is not a standardized but a stochastic process, it has also to be assumed that resistant colonization below the detection limits of the real-time PCR assays will have been overlooked, and so, the true prevalence of resistance-gene abundance might have been even higher.

Due to the same reason, it is likely that several sexually transmitted infections (STI) might have been overlooked by the described screening approach and that the recorded chlamydial and gonococcal infections just indicate the highly replicative ones. Focusing on *Chlamydia trachomatis* and *Neisseria gonorrhoeae*, the recorded prevalence within the low one-digit percentage range is nevertheless similar, like those reported from previous assessments with Ivory Coastal and Ghanian women without selection for high-risk populations [54,109,110,111]. At least for Ghana, these percentages have been relatively stable for decades [109,110,111], while a higher *N. gonorrhoeae* prevalence was reported from Ghanaian military hospitals with a predominance of infected male individuals [112]. As PCR can detect *Treponema pallidum* only from bacteria-containing lesions or the contamination caused by them, the absence of *T. pallidum* DNA above the detection limit as recorded in this study does not necessarily exclude the abundance of active syphilis. Previous studies report serological responses to syphilitic infections in <5% of pregnant Ghanaian women [113] and even lower rates of active infections [114]. Accordingly, the absence of *T. pallidum* DNA in the samples is not unexpected. The lack of abundance of *Mycoplasma-genitalium*-specific DNA is much more difficult to interpret. While *M. genitalium* was the most frequently detected bacterium in Ghanian women attending an STI clinic with a positivity rate of 77.1% in a previous assessment [115], standardized surveillance results from pregnant Ghanaian and Ivory Coastal women in general without selection for STI-specific risk factors are missing so far.

Both *Schistosoma haematobium* and *S. mansoni* are common in Ivory Coast and Ghana with regionally varying prevalence rates [28,29,30,31,32,33,34,35,36,116,117,118,119,120,121,122,123,124,125,126,127,128,129,130,131,132]. Accordingly, the recorded high overall prevalence of about 20 percent is not surprising, while the observed dominance of placental schistosomiasis in Ghana might reflect regional factors. In more detail, the detected particularly high prevalence of genital schistosomiasis in the placental tissues of the Ghanaian women might not be representative for Ghana but might just indicate a high regional prevalence at the specific study site. The predominance of *S. haematobium*-complex DNA in the assessed samples does not necessarily reflect a regionally higher abundance compared to *S. mansoni* complex but simply the fact that *S. haematobium* is the causative agent of urogenital schistosomiasis [24,25], and so, higher quantities of *S. haematobium*-complex DNA can be expected in placenta tissue. The single positive real-time PCR signal for *S. mansoni* complex with a high Ct value, in contrast, most likely indicates aberrant migration in the case of a high worm burden. As expected for urogenital schistosomiasis, this medical condition was associated with increased delivery rates by Caesarian section and a slightly reduced APGAR 1 score value. The recorded associations between possessing a freezer and better sanitary equipment are much more difficult to explain. However, considering the fact that the study was not specifically powered to address such associations, it might be speculated that they were merely by chance and, thus, statistical artifacts. Such phenomena are not uncommon if multiple testing is performed.

When comparing the distribution of bacterial STI-related pathogens, as well as *Schistosoma* spp., with the distribution of resistance genes, one phenomenon seems noteworthy. Most of the pathogen detections were associated either with no or only with a single concomitantly detected beta-lactamase gene. Accordingly, one might assume that there could be a subpopulation with ready access to the consumption of antimicrobial drugs resulting in a selection pressure and associated high colonization rates with resistant microorganisms, while another group with low access to such drugs and, thus, lower beta-lactam-resistant colonization is more likely to be infected with parasites and causative agents of sexually transmitted infections. However, the explorative epidemiological assessment did not suggest any clear association of placental schistosomiasis and limited economic resources. Further, the low case numbers prevented an in-depth assessment of this hypothesis. However, future studies might address this question. If multidrug-resistant colonization is transmitted not only to placenta tissue but to newborns during vaginal delivery and eventually causes infections, antimicrobial-drug-induced selection of resistant vaginal colonization may become a risk factor to consider.

This study has a number of limitations. First, the interpretation of the study results is hampered by the study’s retrospective design and the low number of available samples. The abovementioned die-off of Gram-negative resistant bacteria caused by prolonged sample storage in spite of adequate storage conditions, deep-frozen at −80 °C, impressively confirms the relevance of this limiting factor. Second, the assessment of contaminations on placenta tissue is not a standardized procedure for the screening for resistant bacterial colonization, nor for sexually transmitted infections, and it is definitely not a method of choice for such analyses. Accordingly, the sensitivity of the respective assessments was necessarily lower compared to standardized screening procedures. However, more specific sampling had not been performed, and the investigation of contaminations on vaginally delivered placenta tissue was considered as a surrogate parameter for high pathogen densities associated with a high likeliness of smear-based transmission to the newborn. Third, none of the applied test assays were specifically developed for screenings with placenta tissue, which is a quite unusual screening site for molecular diagnostic approaches in infectious disease medicine. Accordingly, individual false-positive results caused by unexpected reactions in this sample matrix cannot be excluded with definitive certainty. Fourth, the epidemiological associations based on the applied simple statistical calculations shall be considered as hypothesis forming only in this exploratory approach. This cross-sectional study was not powered to specifically address the calculated associations, and so, future specific assessments need to confirm or deny the reproducibility of these observations. Fifth, the real-time PCR-based screening approach did not allow any conclusions on the time of infection or colonization in relation to pregnancy, which is a limitation intrinsic to the chosen methodology. Sixth, considering the low number of samples available for the study, we abstained from planning in-depth assessments of associations of various detected pathogens. In spite of ongoing discussion on, e.g., associations of sexually transmitted infections and genital schistosomiasis [133,134,135], respective assessments were not performed for this reason. Indeed, only a single co-infection of *Chlamydia trachomatis* and *Schistosoma haematobium* complex was recorded, which does not allow any conclusions on this topic, considering the low overall case number.

## 5. Conclusions

In spite of the abovementioned limitations, the assessments have shown high rates of resistant colonization and urogenital schistosomiasis in placenta tissue of pregnant Ghanaian women, next to low to moderate infection rates with *Chlamydia trachomatis* and *Neisseria gonorrhoeae*. As respective colonization events and infections may pose a risk to a pregnancy, as well as to the health of the mother and the newborn, surveillance assessments beyond the here-presented proof-of-principle study seem advisable to minimize the risk by implementing tailored prevention or treatment options.

## Figures and Tables

**Table 1:** Epidemiological information on the assessed placenta samples. Not all data were available for all assessed individuals. The table provides a general overview on the whole Western African study population without stratification by country.

**Country of Origin** (n = 274)	
Ivory Coast (n, %)	223, 81.4%
Ghana (n, %)	51, 18.6%
**Age** (n = 274)	
Mean (±SD)	28.4 (±5.8)
Median (Min., Max.)	28 (18, 46)
**Number of pregnancies** (n = 252)	
Mean (±SD)	3.1 (±2.0)
Median (Min., Max.)	3 (1, 9)
**APGAR 1 score value** (n = 268)	
Mean (±SD)	7.9 (±1.1)
Median (Min., Max.)	8 (2, 10)
**APGAR 2 score value** (n = 268)	
Mean (±SD)	8.7 (±0.7)
Median (Min., Max.)	9 (4, 10)
**Breeding of chicken** (n = 274)	
Yes (n, %)	13.9% (38/274)
No (n, %)	86.1% (236/274)
**Possession of a freezer** (n = 274)	
Yes (n, %)	36.1% (99/274)
No (n, %)	63.9% (175/274)
**Electricity at home** (n = 274)	
Yes (n, %)	98.2% (269/274)
No (n, %)	1.8% (5/274)
**Type of birth** (n = 265)	
Caesarian section (n, %)	7.9% (21/265)
Vaginal delivery (n, %)	92.1% (244/265)
**Floor quality** (n = 274)	
Earth/sand	5.5% (15/274)
Wooden/bamboo	0.0% (0/274))
Vinyl/tiles	18.2% (50/274)
Cement	76.3% (209/274)
Other	0.0% (0/274)
**Education level** (n = 274)	
None	39.8% (109/274)
Basic	29.6% (81/274)
Secondary	22.3% (61/274)
Tertiary	8.4% (23/274)
**Occupation** (n = 274)	
Housewife	21.2% (58/274)
Farmer	0.4% (1/274)
Trader	21.5% (59/274)
Salary worker	10.9% (30/274)
Other	46.0% (126/274)
**Drinking water source** (n = 274)	
Surface water	0.0% (0/274)
Tanker	0.4% (1/274)
Well	3.3% (9/274)
Bore hole	0.0% (0/274)
Piped water	94.9% (260/274)
Other	1.5% (4/274)
**Toilet** (n = 274)	
No facility	0.0% (0/274)
Pit latrine	54.0% (148/274)
Improved pit latrine	26.6% (73/274)
Flush toilet	19.3% (53/274)
Other	0.0% (0/274)

n = number. % = percent. SD = standard deviation. Min. = minimum. Max. = maximum. APGAR = appearance, pulse, grimace, activity and respiration.

**Table 2:** Molecular detection of resistance genes within the samples.

PCR Target	Numbers and Proportions of Detections n/n (%)	Mean (SD) of the Measured CT Values	Median (Min., Max.) of the Measured CT Values
*bla*_CTX-M_	42/268 (15.7%)	30.9 (2.8)	32 (24, 37)
*bla*_NDM_	6/268 (2.2%)	29.5 (1.9)	30 (27, 33)
*bla*_KPC_	2/268 (0.7%)	31.0 (1.0)	31 (30, 32)
*bla*_VIM_	12/268 (4.5%)	30.0 (3.1)	31 (21, 33)
*bla*_IMP_	25/268 (9.3%)	30.4 (2.0)	30 (26, 34)
*bla*_OXA-23_-like	5/268 (1.9%)	30.8 (4.5)	32 (24, 37)
*bla*_OXA-40/24_-like	0/268 (0%)	n.e.	n.e.
*bla*_OXA-48_-like	5/268 (1.9%)	31.8 (2.8)	31 (29, 37)
*bla*_OXA-58_-like	10/268 (3.7%)	34.2 (3.3)	35.5 (26, 37)
*bla*_GES_	20/268 (7.5%)	35.2 (3.2)	35 (29, 40)
*bla*_NMC_A/IMI_	0/268 (0%)	n.e.	n.e.
*bla*_BIC_	0/268 (0%)	n.e.	n.e.
*bla*_SME_	0/268 (0%)	n.e.	n.e.
*bla*_GIM_	0/268 (0%)	n.e.	n.e.
*bla*_DIM_	0/268 (0%)	n.e.	n.e.

PCR = polymerase chain reaction. SD = standard deviation. CT = cycle threshold. Min. = minimum. Max. = maximum. n.e. = not estimable.

**Table 3:** Molecular detection of causative agents of sexually transmitted infections within the samples.

PCR Target	Numbers and Proportions of Detections n/n (%)	Mean (SD) of the Measured CT Values	Median (Min., Max.) of the Measured CT Values
*Treponema pallidum*	0/268 (0%)	n.e.	n.e.
*Chlamydia trachomatis* screening	3/268 (1.1%)	31.3 (2.6)	30 (29, 35)
*Chlamydia trachomatis* differentiation: LGV-associated	0/268 (0%)	n.e.	n.e.
*Chlamydia trachomatis* differentiation: non-LGV-associated	3/268 (1.1%)	34.3 (1.7)	35 (32, 36)
*Mycoplasma genitalium*	0/268 (0%)	n.e.	n.e.
*Neisseria gonorrhoeae* *	5/268 (1.9%)	Please see below!	Please see below!
multi-copy *opa* genes	5/268 (1.9%)	31.8 (2.2)	33 (29, 34)
*porA* pseudogene	5/268 (1.9%)	34.0 (2.6)	33 (31, 38)

PCR = polymerase chain reaction. SD = standard deviation. CT = cycle threshold. Min. = minimum. Max. = maximum. n.e. = not estimable. LGV = lymphogranuloma venereum. * In two further instances, *N. gonorrhoeae* could not be confirmed because of negative *porA*-pseudogene PCR in spite of positive *opa* gene PCR with Ct values of 32 and 34, respectively.

**Table 4:** Molecular detection of *Schistosoma mansoni* complex and *Schistosoma haematobium* complex within the samples.

PCR Target	Numbers and Proportions of Detections n/n (%)	Mean (SD) of the Measured CT Values	Median (Min., Max.) of the Measured CT Values
*Schistosoma mansoni* complex	1/268 (0.4%)	37 (n.a.)	37 (n.a.)
*Schistosoma haematobium* complex	50/269 * (18.6%)	31.0 (1.6)	32 (27, 34)

PCR = polymerase chain reaction. SD = standard deviation. CT = cycle threshold. Min. = minimum. Max. = maximum. n.a. = not applicable. * The denominator is 269 in this case because a positive real-time PCR result was observed even in a formally inhibited sample, and so, this sample was included for this particular assessment.

## Data Availability

All relevant data are provided in the manuscript and Appendix A. Raw data can be made available on reasonable request.

## Appendix A

**Table A1:** Target genes, calculated detection limits and oligonucleotides used for the resistance-gene-specific and pathogen-specific real-time PCR assays. Hyphens in the oligonucleotide sequences have been inserted to increase the readability, not to delineate codon triplets.

PCR Target	CTX-M-Type Beta-Lactamase (Groups I–V)
Target gene	*bla*_CTX-M_
Detection limit	<10^2^ copies/µL
Forward primer I	5′-GCT-GGA-CTG-CCT-GCT-TCC-T-3′
Forward primer II	5′-TGC-CGA-AAT-CAT-GGG-TAG-TG-3′
Forward primer III	5′-CTA-CCC-ACA-TCG-TGG-GTT-GTC-3′
Forward primer IV/V	5′-ATT-CGG-GCC-GGC-TTA-CC-3′
Reverse primer I	5′-CGT-TGG-TGG-TGC-CAT-AGY-CA-3′
Reverse primer II	5′-TCG-TTG-GTG-GTG-CCA-TAA-TCT-3′
Reverse primer III	5′-GAT-GTC-ATT-CGT-CGT-ACC-ATA-ATC-A-3′
Reverse primer IV	5′-ATC-ATT-GGT-GGT-GCC-GTA-GYC-3′
Reverse primer V	5′-GCG-ATA-TCA-TTC-GTC-GTA-CCA-TAA-3′
Probe and modifications	5‘-VIC-CCG-CTG-CCG-GTC-TTA-TC-MGB-NFQ-3‘
Positive control plasmid insert	5′-CGC-AGC-CAG-CAT-TCG-GGC-CGG-CTT-ACC-GAC-GTC-GTG-GAC-TGT-GGG-TGA-TAA-GAC-CGG-CAG-CGG-CGA-CTA-CGG-CAC-CAC-CAA-TGA-TAT-TGC-GGT-GA-3′
GenBank accession number of the insert	OM355481.1
Reference	[59]
PCR target	VIM-type beta-lactamase
Target gene	*bla*_VIM_
Detection limit	<10^2^ copies/µL
Forward primer	5′-GAG-ATT-CCC-ACG-CAY-TCT-CTA-GA-3′
Reverse primer	5′-AAT-GCG-CAG-CAC-CAG-GAT-AG-3′
Probe and modifications	5′-JOE-ACG-CAG-TGC-GCT-TCG-GTC-CAG-T-BHQ1-3′
Positive control plasmid insert	5′-AGA-GGG-GAG-CGA-GAT-TCC-CAC-GCA-CTC-TCT-AGA-AGG-ACT-CTC-ATC-GAG-CGG-GGA-CGC-AGT-GCG-CTT-CGG-TCC-AGT-AGA-ACT-CTT-CTA-TCC-TGG-TGC-TGC-GCA-TTC-GAC-CGA-CAA-3′
GenBank accession number of the insert	NG_050338.1
Reference	[59]
PCR target	IMP-type beta-lactamase
Target gene	*bla*_IMP_
Detection limit	<10^2^ copies/µL
Forward primer	5′-GGC-GGA-ATA-GAG-TGG-CTT-AAT-TCT-C-3′
Reverse primer I	5′-GAA-TTT-TTA-GCT-TGT-ACT-TTA-CCG-TCT-TT-3′
Reverse primer II	5′-ATT-TTT-AGC-TTG-TAC-CTT-ACC-GTA-TT-3′
Reverse primer III	5′-TTT-GTA-GCT-TGC-ACC-TTA-TTG-TCT-TT-3′
Probe I and modifications	5′-FAM-ATG-CAT-CTG-AAT-TAA-C-MGB-TAMRA-3′
Probe II and modifications	5‘-FAM-TAT-*GCA-TCT-*GAA-T*TA-A*CA-AAT-*GA-TAMRA-3‘
Positive control plasmid insert	5′-CGA-CAG-CAC-GGG-CGG-AAT-AGA-GTG-GCT-TAA-TTC-TCA-ATC-TAT-CCC-CAC-GTA-TGC-ATC-TGA-ATT-AAC-AAA-TGA-ACT-TCT-TAA-AAA-AGA-CGG-TAA-AGT-ACA-AGC-TAA-AAA-TTC-ATT-TAG-CGG-AG-3′
GenBank accession number of the insert	NG_049212.1
Reference	[59]
PCR target	NDM-type beta-lactamase
Target gene	*bla*_NDM_
Detection limit	<10^2^ copies/µL
Forward primer	5′-CAT-TAG-CCG-CTG-CAT-TGA-TG-3′
Reverse primer	5′-GTC-GCC-AGT-TTC-CAT-TTG-CT-3′
Probe and modifications	5′-ROX-CAT-GCC-CGG-TGA-AAT-CCG-CC-BHQ2-3′
Positive control plasmid insert	5′-CTG-AGC-ACC-GCA-TTA-GCC-GCT-GCA-TTG-ATG-CTG-AGC-GGG-TGC-ATG-CCC-GGT-GAA-ATC-CGC-CCG-ACG-ATT-GGC-CAG-CAA-ATG-GAA-ACT-GGC-GAC-CAA-CGG-TTT-GGC-3′
GenBank accession number of the insert	NG_088409.1
Reference	[59]
PCR target	KPC-type beta-lactamase
Target gene	*bla*_KPC_
Detection limit	<10^2^ copies/µL
Forward primer	5′-TGC-AGA-GCC-CAG-TGT-CAG-TTT-3′
Reverse primer	5′-CGC-TCT-ATC-GGC-GAT-ACC-A-3′
Probe and modifications	5′-Cy5-TTC-CGT-CAC-GGC-GCG-CG-BHQ2-3′
Positive control plasmid insert	5′-GGC-CTT-CAT-GCG-CTC-TAT-CGG-CGA-TAC-CAC-GTT-CCG-TCT-GGA-CCG-CTG-GGA-GCT-GGA-GCT-GAA-CTC-CGC-CAT-CCC-AGG-CGA-TGC-GCG-CTA-TAC-CTC-ATC-GCC-GCG-CGC-CGT-GAC-GGA-AAG-CTT-ACA-AAA-ACT-GAC-ACT-GGG-CTC-TGC-ACT-GGC-TGC-GC-3′
GenBank accession number of the insert	NG_067225.1
Reference	[59]
PCR target	OXA-23-like-type beta-lactamase
Target gene	*bla*_OXA-23_-like
Detection limit	<10^2^ copies/µL
Forward primer	5′-TAA-ATG-GAA-GGG-CGA-GAA-3′
Reverse primer	5′-ACC-TGC-TGT-CCA-ATT-TCA-G-3′
Probe and modifications	5′-FAM-CCA-TGA-AGC-TTT-CTG-CAG-TCC-CAG-TC-TAMRA-3′
Positive control plasmid insert	5′-ATG-AAA-TAT-TTA-AAT-GGA-AGG-GCG-AGA-AAA-GGT-CAT-TTA-CCG-CTT-GGG-AAA-AAG-ACA-TGA-CAC-TAG-GAG-AAG-CCA-TGA-AGC-TTT-CTG-CAG-TCC-CAG-TCT-ATC-AGG-AAC-TTG-CGC-GAC-GTA-TCG-GTC-TTG-ATC-TCA-TGC-AAA-AAG-AAG-TAA-AAC-GTAT-TGG-TTT-CGG-TAA-TGC-TGA-AAT-TGG-ACA-GCA-GGT-TGA-TAA-TTT-C-3′
GenBank accession number of the insert	OM310935.1
Reference	[60]
PCR target	OXA-40/24-like-type beta-lactamase
Target gene	*bla*_OXA-40/24_-like
Detection limit	<10^2^ copies/µL
Forward primer	5′-TGA-CTT-TAG-GTG-AGG-CAA-TG-3′
Reverse primer	5′-GTT-ATG-TGC-AAG-GTC-ATC-GG-3′
Probe and modifications	5′-Cy5-TGC-AAG-ACG-GAC-TGG-CCT-AGA-GCT-AAT-BHQ2-3′
Positive control plasmid insert	5′-GAG-AAA-GAT-ATG-ACT-TTA-GGT-GAG-GCA-ATG-GCA-TTG-TCA-GCA-GTT-CCA-GTA-TAT-CAA-GAG-CTT-GCA-AGA-CGG-ACT-GGC-CTA-GAG-CTA-ATG-CAG-AAA-GAA-GTA-AAG-CGG-GTT-AAT-TTT-GGA-AAT-ACA-AAT-ATT-GGA-ACA-CAG-GTC-GAT-AAT-TTT-TGG-TTA-GTT-GGC-CCC-CTT-AAA-ATT-ACA-CCA-GTA-CAA-GAA-GTT-AAT-TTT-GCC-GAT-GAC-CTT-GCA-CAT-AAC-CGA-TTA-CCT-T-3′
GenBank accession number of the insert	NG_078047.1
Reference	[60]
PCR target	OXA-48-like-type beta-lactamase
Target gene	*bla*_OXA-48_-like
Detection limit	<10^2^ copies/µL
Forward primer	5′-AGG-GCG-TAG-TTG-TGC-TC-3′
Reverse primer	5′-GTG-TTC-ATC-CTT-AAC-CAC-GC-3′
Probe and modifications	5′-ROX-TCT-TAA-ACG-GGC-GAA-CCA-AGC-AT-BHQ2-3′
Positive control plasmid insert	5′-CAT-AAA-TCA-CAG-GGC-GTA-GTT-GTG-CTC-TGG-AAT-GAG-AAT-AAG-CAG-CAA-GGA-TTT-ACC-AAT-AAT-CTT-AAA-CGG-GCG-AAC-CAA-GCA-TTT-TTA-CCC-GCA-TCT-ACC-TTT-AAA-ATT-CCC-AAT-AGC-TTG-ATC-GCC-CTC-GAT-TTG-GGC-GTG-GTT-AAG-GAT-GAA-CAC-CAA-GTC-TTT-A-3′
GenBank accession number of the insert	ON651448.1
Reference	[60]
PCR target	OXA-58-like-type beta-lactamase
Target gene	*bla*_OXA-58_-like
Detection limit	<10^2^ copies/µL
Forward primer	5′-ATT-GGC-ACG-TCG-TAT-TGG-3′
Reverse primer	5′-CCC-CTC-TGC-GCT-CTA-CAT-A-3′
Probe and modifications	5′-JOE-AGT-GAA-TTG-CAA-CGT-ATT-GGT-TAT-GGC-A-BHQ1-3′
Positive control plasmid insert	5′-TAT-ATC-AAG-AAT-TGG-CAC-GTC-GTA-TTG-GTC-CAA-GCT-TAA-TGC-AAA-GTG-AAT-TGC-AAC-GTA-TTG-GTT-ATG-GCA-ATA-TGC-AAA-TAG-GCA-CGG-AAG-TTG-ATC-AAT-TTT-GGT-TGA-AAG-GGC-CTT-TGA-CAA-TTA-CAC-CTA-TAC-AAG-AAG-TAA-AGT-TTG-TGT-ATG-ATT-TAG-CCC-AAG-GGC-AAT-TGC-CTT-TTA-AAC-CTG-AAG-TTC-AGC-AAC-AAG-TGA-AAG-AGA-TGT-TGT-ATG-TAG-AGC-GCA-GAG-GGG-AGA-ATC-GTC-T-3′
GenBank accession number of the insert	KY660721.1
Reference	[60]
PCR target	GES-type beta-lactamase
Target gene	*bla*_GES_
Detection limit	<10^2^ copies/µL
Forward primer	5′-TGG-CTA-AAG-TCC-TCT-ATG-3′
Reverse primer	5′-CAA-CCC-AAT-CTT-TAG-GAA-A-3′
Probe and modifications	5′-FAM-CGT-CTC-CCG-TTT-GGT-TTC-CG-TAMRA-3′
Positive control plasmid insert	5′-GCA-CGT-ACT-GTG-GCT-AAA-GTC-CTC-TAT-GGC-GGC-GCA-CTG-ACG-TCC-ACC-TCG-ACC-CAC-ACC-ATT-GAG-AGG-TGG-CTG-ATC-GGA-AAC-CAA-ACG-GGA-GAC-GCG-ACA-CTA-CGA-GCG-GGT-TTT-CCT-AAA-GAT-TGG-GTT-GTT-GGA-GAG-AA-3′
GenBank accession number of the insert	NG_080773.1
Reference	[61]
PCR target	NMC-A/IMI-type beta-lactamase
Target gene	*bla*_NMC-A/IMI_
Detection limit	<10^2^ copies/µL
Forward primer	5′-GTC-ACT-TAA-TGT-AAA-ACC-AA-3′
Reverse primer	5′-CTA-CCA-TTG-AAA-TCT-GTT-TC-3′
Probe and modifications	5′-Cy5-AGC-CAT-CTT-GTT-TAG-CTC-TTG-TTT-AGT-BHQ2-3′
Positive control plasmid insert	5′-ATG-TCA-CTT-AAT-GTA-AAA-CCA-AGC-AGA-ATA-GCC-ATC-TTG-TTT-AGC-TCT-TGT-TTA-GTT-TCA-ATA-TCA-TTT-TTC-TCA-CAG-GCC-AAT-ACA-AAG-GGC-ATC-GAT-GAT-ATT-AAA-AAC-CTT-GAA-ACA-GAT-TTC-AAT-GGT-AGA-ATT-GGT-GTC-3′
GenBank accession number of the insert	NG_065426.1
Reference	[61]
PCR target	BIC-type beta-lactamase
Target gene	*bla*_BIC_
Detection limit	<10^2^ copies/µL
Forward primer	5′-GGA-GAA-ACG-TAT-CGA-CTA-TA-3′
Reverse primer	5′-TCC-AGA-AGC-AAA-TTT-GTC-3′
Probe and modifications	5′-JOE-CAC-CGT-TGT-CGC-TGT-ACT-GC-BHQ1-3′
Positive control plasmid insert	5′-AAG-GCT-TAC-TGG-AGA-AAC-GTA-TCG-ACT-ATA-AGA-ATC-GGG-TGA-TGG-AAC-CTC-ACT-CTC-CCA-TCA-GCG-CAC-AAC-ATA-GTT-CGA-CGG-GTA-TGA-CCG-TGG-CGC-AAT-TAG-CGG-CAG-CGG-CGC-TGC-AGT-ACA-GCG-ACA-ACG-GTG-CGA-CAA-ATT-TGC-TTC-TGG-AAA-ACG-TTC-TG-3′
GenBank accession number of the insert	NG_048708.1
Reference	[61]
PCR target	SME-type beta-lactamase
Target gene	*bla*_SME_
Detection limit	<10^2^ copies/µL
Forward primer	5′-GGC-TCA-GGT-ATG-ACA-TTA-3′
Reverse primer	5′-TCT-CCA-ATA-GAA-CGC-ATA-A-3′
Probe and modifications	5′-ROX-CTC-AGG-ACC-GCC-AAG-AAA-TCG-BHQ2-3′
Positive control plasmid insert	5′-AAA-ATA-TAA-AGG-CTC-AGG-TAT-GAC-ATT-AGG-TGA-TAT-GGC-TTC-TGC-TGC-ATT-GCA-ATA-TAG-CGA-CAA-TGG-GGC-AAC-AAA-TAT-AAT-TAT-GGA-ACG-ATT-TCT-TGG-CGG-TCC-TGA-GGG-GAT-GAC-TAA-ATT-TAT-GCG-TTC-TAT-TGG-AGA-TAA-TGA-GTT-T-3′
GenBank accession number of the insert	MN182491.1
Reference	[61]
PCR target	GIM-type beta-lactamase
Target gene	*bla*_GIM_
Detection limit	<10^2^ copies/µL
Forward primer	5′-TCG-ACA-CAC-CTT-GGT-CTG-AA-3′
Reverse primer	5′-AAC-TTC-CAA-CTT-TGC-CAT-GC-3′
Probe and modifications	5′-FAM-CAC-GAA-GTT-GTT-ATT-ATC-CTG-GGC-GAC-T-TAMRA-3′
Positive control plasmid insert	5′-GCC-TAT-ATT-ATC-GAC-ACA-CCT-TGG-TCT-GAA-GAA-GAC-ACG-AAG-TTG-TTA-TTA-TCC-TGG-GCG-ACT-GAC-AGG-GGA-TAC-CAG-GTT-ATG-GCT-AGC-ATC-TCA-ACT-CAT-TCT-CAT-GGA-GAT-CGC-ACT-GCT-GGT-ATC-AAG-TTG-CTA-AAT-TCA-AAG-TCA-ATT-CCT-ACA-TAC-ACA-TCA-GAG-TTA-ACT-AAA-AAG-CTT-CTT-GCC-CGT-GAA-GGA-AAG-CCG-GTT-CCT-ACC-CAC-TAC-TTT-AAA-GAC-GAC-GAA-TTC-ACA-CTG-GGA-AAT-GGG-CTT-ATA-GAG-CTC-TAC-TAT-CCA-GGT-GCT-GGG-CAT-ACA-GAG-GAT-AAT-ATT-GTT-GCT-TGG-TTA-CCC-AAA-AGC-AAA-ATA-CTA-TTT-GGT-GGC-TGC-CTC-GTG-AGG-AGT-CAT-GAG-TGG-GAA-GGC-TTA-GGT-TAC-GTA-GGC-GAC-GCC-TCA-ATT-AGC-TCT-TGG-GCT-GAC-TCA-ATT-AAA-AAT-ATT-GTA-TCG-AAA-AAA-TAT-CCC-ATT-CAA-ATG-GTC-GTT-CCG-GGG-CAT-GGC-AAA-GTT-GGA-AGT-TCA-GAT-ATA-TT-3′
GenBank accession number of the insert	MK847892.1
Reference	[62], probe from this study
PCR target	DIM-type beta-lactamase
Target gene	*bla*_DIM_
Detection limit	<10^2^ copies/µL
Forward primer	5′-GCT-TGT-CTT-CGC-TTG-CTA-ACG-3′
Reverse primer	5′-CGT-TCG-GCT-GGA-TTG-ATT-TG-3′
Probe and modifications	5′-Cy5-ACA-CAT-CAT-ACA-GTC-GTG-TGA-ATG-GGT-TTG-BHQ2-3′
Positive control plasmid insert	5′-CTT-CTA-TTC-AGC-TTG-TCT-TCG-CTT-GCT-AAC-GAC-GAG-GTA-CCT-GAG-CTA-AGA-ATC-GAG-AAA-GTA-AAA-GAG-AAC-ATC-TTT-TTG-CAC-ACA-TCA-TAC-AGT-CGT-GTG-AAT-GGG-TTT-GGT-TTG-GTC-AGT-TCA-AAC-GGC-CTT-GTT-GTC-ATA-GAT-AAG-GGT-AAT-GCT-TTC-ATT-GTT-GAT-ACA-CCT-TGG-TCA-GAC-CGA-GAT-ACA-GAA-ACG-CTC-GTA-CAT-TGG-ATT-CGT-AAA-AAT-GGT-TAT-GAG-CTA-CTG-GGG-AGT-GTT-TCT-ACT-CAT-TGG-CAT-GAG-GAT-AGA-ACC-GCA-GGA-ATT-AAA-TGG-CTT-AAT-GAC-CAA-TCA-ATT-TCT-ACG-TAT-GCC-ACG-ACT-TCA-ACC-AAC-CAT-CTC-TTG-AAA-GAA-AAT-AAA-AAA-GAG-CCA-GCG-AAA-TAC-ACC-TTG-AAA-GGA-AAT-GAG-TCC-ACA-TTG-GTT-GAC-GGC-CTT-ATC-GAA-GTA-TTT-TAT-CCA-GGA-GGT-GGT-CAT-ACA-ATA-GAC-AAC-GTA-GTG-GTG-TGG-TTG-CCA-AAG-TCG-AAA-ATC-TTA-TTT-GGC-GGC-TGT-TTT-GTG-CGT-AGC-CTT-GAT-TCC-GAG-GGG-TTA-GGC-TAC-ACT-GGT-GAA-GCC-CAT-ATT-GAT-CAA-TGG-TCC-CGA-TCA-GCT-CAG-AAT-GCT-CTG-TCT-AGG-TAC-TCA-GAA-GCC-CAG-ATA-GTA-ATT-CCT-GGC-CAT-GGG-AAA-ATC-GGG-GAT-ATA-GCG-CTG-TTA-AAA-CAC-ACC-AAA-AGT-CTG-GCT-GAG-ACA-GCC-TCT-AAC-AAA-TCA-ATC-CAG-CCG-AAC-GCT-AAC-GCG-TC-3′
GenBank accession number of the insert	NG_049077.1
Reference	[62], probe from this study
PCR target	*Treponema pallidum*
Target gene	*polA*
Detection limit	<10^2^ copies/µL
Forward primer	5′-AGG-ATC-CGG-CAT-ATG-TCC-AA-3′
Reverse primer	5′-GTG-AGC-GTC-TCA-TCA-TTC-CAA-A-3′
Probe and modifications	5′-FAM-ATG-CAC-CAG-CTT-CGA-MGB-NFQ-3′
Positive control plasmid insert	5′-TCT-GCT-GTG-CAG-GAT-CCG-GCA-TAT-GTC-CAA-GCT-GTC-ATG-CAC-CAG-CTT-CGA-CGT-CTT-TGG-AAT-GAT-GAG-ACG-CTC-ACA-CTT-GTT-ATG-3′
GenBank accession number of the insert	U57757.1
Reference	[63]
PCR target	*Chlamydia trachomatis* (screening)
Target gene	*Chlamydia trachomatis* cryptic plasmid sequence
Detection limit	<10^2^ copies/µL
Forward primer	5′-GGA-TTG-ACT-CCG-ACA-ACG-TAT-TC-3′
Reverse primer	5′-ATC-ATT-GCC-ATT-AGA-AAG-GGC-ATT-3′
Probe and modifications	5′-Cy5-TTA-CGT-GTA-GGC-GGT-TTA-GAA-AGC-GG-BHQ2-3′
Positive control plasmid insert	5′-TAC-TAA-TAC-AGG-ATT-GAC-TCC-GAC-AAC-GTA-TTC-ATT-ACG-TGT-AGG-CGG-TTT-AGA-AAG-CGG-TGT-GGT-ATG-GGT-TAA-TGC-CCT-TTC-TAA-TGG-CAA-TGA-TAT-TTT-AGG-AA-3′
GenBank accession number of the insert	CP010570.1
Reference	[64]
PCR target	*Chlamydia trachomatis* (differentiation)
Target gene	*pmpH*
Detection limit	<10^2^ copies/µL
Forward primer	5′-GGA-TAA-CTC-TGT-GGG-GTA-TTC-TCC-T-3′
Reverse primer	5′-AGA-CCC-TTT-CCG-AGC-ATC-ACT-3′
Probe and modifications (pan-serovar)	5′-FAM-CCT-GCT-CCA-ACA-GT-MGB-NFQ-3′
Probe and modifications (A-K-serovars only)	5′-ROX-GCT-TGA-AGC-AGC-AGG-AGC-TGG-TG-BHQ2-3′
Positive control plasmid insert (pan-serovar)	5′-TTG-ATT-TTC-TGG-GAT-AAC-TCC-GTG-GGG-TAT-TCT-CCT-TTA-TCT-ACT-GTG-CCA-ACC-TCA-TCA-TCA-ACT-CCG-CCT-GCT-CCA-ACA-GTT-AGT-GAT-GCT-CGG-AAA-GGG-TCT-ATT-TTT-TCT-G-3′
GenBank accession number of the insert	AY184168.1
Positive control plasmid insert (A-K-serovars only)	5′-GTG-ATT-TTT-TGG-GAT-AAC-TCT-GTG-GGG-TAT-TCT-CCT-TTG-TCT-ATT-GTG-CCA-GCA-TCG-ACT-CCA-ACT-CCT-CCA-GCA-CCA-GCA-CCA-GCT-CCT-GCT-GCT-TCA-AGC-TCT-TTA-TCT-CCA-ACA-GTT-AGT-GAT-GCT-CGG-AAA-GGG-TCT-ATT-TTT-TCT-G-3′
GenBank accession number of the insert	AY184158.1
Reference	[64]
PCR target	*Mycoplasma genitalium*
Target gene	sequence of the MgPA operon
Detection limit	<10^2^ copies/µL
Forward primer	5′-GAG-AAA-TAC-CTT-GAT-GGT-CAG-CAA-3′
Reverse primer	5′-GTT-AAT-ATC-ATA-TAA-AGC-TCT-ACC-GTT-GTT-ATC-3′
Probe and modifications	5‘-ROX-AC*T-TT*G-CAA-*TC*A-*GAA-*GGT-BHQ2-3‘
Positive control plasmid insert	5′-CAA-TGC-TGT-TGA-GAA-ATA-CCT-TGA-TGG-TCA-GCA-AAA-CTT-TGC-AAT-CAG-AAG-GTA-TGA-TAA-CAA-CGG-TAG-AGC-TTT-ATA-TGA-TAT-TAA-CTT-AGC-AAA-AA-3′
GenBank accession number of the insert	M31431.1
Reference	[65,66]
PCR target	*Neisseria gonorrhoeae* (PCR 1 out of 2)
Target gene	multi-copy *opa* genes
Detection limit	<10^2^ copies/µL
Forward primer	5′-TTG-AAA-CAC-CGC-CCG-GAA-3′
Reverse primer	5′-TTT-CGG-CTC-CTT-ATT-CGG-TTT-AA-3′
Probe and modifications	5′-JOE-CCG-ATA-TAA-TC*C-GTC-*CTT-CAA-*CAT-CAG-BHQ1-3′
Positive control plasmid insert	5′-CCA-TAT-TGT-GTT-GAA-ACA-CCG-CCC-GGA-ACC-CGA-TAT-AAT-CCG-TCC-TTC-AAC-ATC-AGT-GAA-AAT-CTT-TTT-TTA-ACC-GGT-TAA-ACC-GAA-TAA-GGA-GCC-GAA-AAT-GAA-TCC-AG-3′
GenBank accession number of the insert	X52372.1
Reference	[67]
PCR target	*Neisseria gonorrhoeae* (PCR 2 out of 2)
Target gene	*porA* pseudogene
Detection limit	<10^2^ copies/µL
Forward primer	5′-CAG-CAT-TCA-ATT-TGT-TCC-GAG-TC-3′
Reverse primer	5′-GAA-CTG-GTT-TCA-TCT-GAT-TAC-TTT-CCA-3′
Probe and modifications	5′-Cy5-CGC-CTA-TAC-GCC-TGC-TAC-TTT-CAC-GC-BHQ2-3′
Positive control plasmid insert	5′-GTT-TCA-GCG-GCA-GCA-TTC-AAT-TTG-TTC-CGA-GTC-AAA-ACA-GCA-AGT-CCG-CCT-ATA-CGC-CTG-CTA-CTT-TCA-CGC-TGG-AAA-GTA-ATC-AGA-TGA-AAC-CAG-TTC-CGG-CTG-TTG-T-3′
GenBank accession number of the insert	AJ010732.1
Reference	[67]
PCR target	*Schistosoma haematobium* complex
Target gene	*Dra1* multi-copy sequence
Detection limit	5 × 10^2^ copies/µL
Forward primer	5′-GAT-CTC-ACC-TAT-CAG-ACG-AAA-C-3′
Reverse primer	5′-TCA-CAA-CGA-TAC-GAC-CAA-C-3′
Probe and modifications	5′-JOE-TGT-TGG-TGG-AAG-GCC-TGT-TTG-CAA-BHQ1-3′
Positive control plasmid insert	5‘-AAA-TTG-GAT-CTC-ACC-TAT-CAG-ACG-AAA-CAA-AGA-AAA-TTT-TAA-AAT-TGT-TGG-TGG-AAG-TGC-CTG-TTT-CGC-AAT-ATC-TCC-GGA-ATG-GTT-GGT-CGT-ATC-GTT-GTG-AAA-ATT-G-3‘
GenBank accession number of the insert	DQ157698.1
Reference	[68]
PCR target	*Schistosoma mansoni* complex
Target gene	*Sm1-7* multi-copy sequence
Detection limit	5 × 10^2^ copies/µL
Forward primer	5′-CCA-CGC-TCT-CGC-AAA-TAA-TCT-3′
Reverse primer	5′-CAA-CCG-TTC-TAT-GAA-AAT-CGT-TGT-3′
Probe and modifications	5′-FAM-TCC-GAA-ACC-ACT-GGA-CGG-ATT-TTT-ATG-AT-BHQ1-3′
Positive control plasmid insert	5‘-TCC-GAC-CAA-CCG-TTC-TAT-GAA-AAT-CGT-TGT-ATC-TCC-GAA-ACC-ACT-GGA-CGG-ATT-TTT-ATG-ATG-TTT-GTT-TTA-GAT-TAT-TTG-CGA-GAG-CGT-GGG-CGT-TA-3‘
GenBank accession number of the insert	M61098.1
Reference	[68]

MGB = minor groove binding, * = subsequent base is locked nucleic acid (LNA).

**Table A2:** Reaction mixes and run conditions for the beta-lactamase real-time PCR assays, part 1 out of 2.

	*bla*_CTX-M_ Gene-Specific Assay	*bla*_VIM_, *bla*_IMP_, *bla*_NDM_ and *bla*_KPC_ Gene-Specific Assay	*bla*_OXA-23_-like, *bla*_OXA-40/24_-like, *bla*_OXA-48_-like and *bla*_OXA-58_-like Gene-Specific Assay
Reaction chemistry			
Master Mix	HotStarTaq (Qiagen)	HotStarTaq (Qiagen)	HotStarTaq (Qiagen)
Reaction volume (µL)	20.0	20.0	20.0
Forward primer concentration (nM)	320.0 (each)	750.0 (each)	800.0 (OXA-48-like & OXA-40/24-like), 600.0 (OXA-58-like), 400.0 (OXA-48-like)
Reverse primer concentration (nM)	320.0 (each)	375 (IMP reverse primers I & II), 750 (all others)	800.0 (OXA-48-like & OXA-40/24-like), 600.0 (OXA-58-like), 400.0 (OXA-48-like)
Probe concentration (nM)	160.0	188.0 (VIM), 200.0 (both IMP probes), 250 (NDM & KPC)	400.0 (OXA-48-like & OXA-40/24-like), 250.0 (OXA-58-like), 150.0 (OXA-48-like)
Final Mg^2+^ concentration (nM)	3.0	3.0	6.0
Bovine serum albumin (ng/µL)	2.0	2.0	2.0
Run conditions			
Initial denaturation	95 °C, 15 min	95 °C, 15 min	95 °C, 15 min
Cycle numbers	40	40	40
Denaturation	95 °C, 15 s	95 °C, 15 s	95 °C, 15 s
Annealing	Combined with amplification	Combined with amplification	Combined with amplification
Amplification	60 °C, 60 s	60 °C, 60 s	60 °C, 60 s
Hold	40 °C, 20 s	40 °C, 20 s	40 °C, 20 s

**Table A3:** Reaction mixes and run conditions for the beta-lactamase real-time PCR assays, part 2 out of 2.

	*bla*_GES_, *bla*_NMC_A/IMI_, *bla*_BIC_ and *bla*_SME_ Gene-Specific Assay	*bla*_GIM_ Gene-Specific Assay	*bla*_DIM_ Gene-Specific Assay
Reaction chemistry			
Master Mix	HotStarTaq (Qiagen)	HotStarTaq (Qiagen)	HotStarTaq (Qiagen)
Reaction volume (µL)	20.0	20.0	20.0
Forward primer concentration (nM)	750.0 (each)	400	400
Reverse primer concentration (nM)	750.0 (each)	400	400
Probe concentration (nM)	375 (NMC_A/IMI), 188 (all others)	200	250
Final Mg^2+^ concentration (nM)	3.0	6.0	6.0
Bovine serum albumin (ng/µL)	2.0	2.0	2.0
Run conditions			
Initial denaturation	95 °C, 15 min	95 °C, 15 min	95 °C, 15 min
Cycle numbers	40	40	40
Denaturation	95 °C, 15 s	95 °C, 30 s	95 °C, 30 s
Annealing	Combined with amplification	46 °C, 40 s	46 °C, 40 s
Amplification	60 °C, 60 s	72 °C, 50 s	72 °C, 50 s
Hold	40 °C, 20 s	40 °C, 20 s	40 °C, 20 s

**Table A4:** Reaction mixes and run conditions for the sexually transmitted infections PCRs and *Schistosoma* spp.-specific PCR assays.

	*Chlamydia trachomatis* Screening and Differentiation Assay	*Neisseria gonorrhoeae*, *Mycoplasma genitalium* and *Treponema pallidum* Assay	*Schistosoma haematobium* Complex and *Schistosoma mansoni* Complex Assay
Reaction chemistry			
Master Mix	HotStarTaq (Qiagen)	HotStarTaq (Qiagen)	HotStarTaq (Qiagen)
Reaction volume (µL)	20.0	20.0	20.0
Forward primer concentration (nM)	50.0 (screening), 600.0 (differentiation)	900.0 (*T. pallidum*), 1000.0 (*M. genitalium*), 400.0 (gonococci, both assays)	500.0 (each)
Reverse primer concentration (nM)	100.0 (screening), 600.0 (differentiation)	900.0 (*T. pallidum*), 1000.0 (*M. genitalium*), 400.0 (gonococci, both assays)	500.0 (each)
Probe concentration (nM)	100.0 (screening), 200.0 (differentiation)	250.0 (*T. pallidum*), 225.0 (*M. genitalium*), 160.0 (gonococci, both assays)	300.0 (each)
Final Mg^2+^ concentration (nM)	4.0	5.0	6.0
Bovine serum albumin (ng/µL)	2.0	2.0	2.0
Run conditions			
Initial denaturation	95 °C, 15 min.	95 °C, 15 min.	95 °C, 15 min.
Cycle numbers	45	50	40
Denaturation	95 °C, 15 sec.	95 °C, 15 sec.	95 °C, 15 sec.
Annealing	Combined with amplification	Combined with amplification	Combined with amplification
Amplification	60 °C, 60 sec.	60 °C, 60 sec.	65 °C, 60 sec.
Hold	40 °C, 20 sec.	40 °C, 20 sec.	40 °C, 10 sec.

Min. = minute, sec. = second.

**Table A5:** Associations of numeric epidemiological parameters with recorded positivity for bacterial resistance determinants, sexually transmitted infections and schistosomiasis (calculated applying Mann–Whitney U testing). Nonidentical denominators result from partially incomplete datasets.

	Bacterial Resistance Determinants			Sexually Transmitted Infections			Schistosomiasis		
	Mean (±SD)	Median (Min., Max.)	Significance P	Mean (±SD)	Median (Min., Max.)	Significance P	Mean (±SD)	Median (Min., Max.)	Significance P
Age in years (+)	28.0 (±5.7)	28 (18, 46)	0.31	25.8 (±5.3)	25 (18, 33)	0.18	29.1 (±5.6)	29 (18, 46)	0.37
Age in years (-)	28.6 (±5.8)	28.5 (18, 43)		28.5 (±5.8)	28 (18, 46)		28.3 (±5.8)	28 (18, 44)	
Number of pregnancies (+)	3.2 (±1.9)	3 (1, 9)	0.70	3.5 (±3.0)	2 (1, 9)	1.00	3.2 (±1.9)	3 (1, 8)	0.57
Number of pregnancies (-)	3.1 (±2.0)	3 (1, 9)		3.1 (±1.7)	3 (1, 9)		3.1 (±2.0)	3 (1, 9)	
APGAR 1 (+)	8.0 (±0.9)	8 (6, 10)	0.17	8.1 (±1.1)	8 (6, 9)	0.46	7.5 (±1.1)	8 (5, 10)	0.01
APGAR 1 (-)	7.8 (±1.1)	8 (2, 10)		7.9 (±1.1)	8 (2, 9)		7.9 (±1.1)	8 (2, 10)	
APGAR 2 (+)	8.8 (±0.5)	9 (7, 10)	0.37	9.1 (±0.3)	9 (9, 10)	0.12	8.6 (±0.7)	9 (7, 10)	0.08
APGAR 2 (-)	8.7 (±0.7)	9 (4, 10)		8.7 (±0.7)	9 (4, 10)		8.8 (±0.6)	9 (4, 10)	

SD = standard deviation. Min. = minimum. Max. = maximum. (+) = parameter detected. (-) = parameter not detected. APGAR = appearance, pulse, grimace, activity and respiration.

**Table A6:** Odds ratios of dichotomous parameters for recorded positivity for bacterial resistance determinants, sexually transmitted infections and schistosomiasis (calculated applying Fisher’s exact test with two-sided *p*-values, the approximation of Woolf for the 95%-confidence interval calculation and Yate’s continuity correction). Nonidentical denominators result from partially incomplete datasets.

	Bacterial Resistance Determinants			Sexually Transmitted Infections			Schistosomiasis		
	Proportion of Samples Positive for Bacterial Resistance Determinants in % (n/n)	Odds Ratio (95%-CI)	Significance P	Proportion of Samples Positive for Sexually Transmitted Infections in % (n/n)	Odds Ratio (95%-CI)	Significance P	Proportion of Samples Positive for Schistosomiasis in % (n/n)	Odds Ratio (95%-CI)	Significance P
Breeding of chicken	18.4% (7/38)	0.45 (0.19, 1.07)	0.09	2.6% (1/38)	0.68 (0.08, 5.54)	1.00	15.8% (6/38)	0.80 (0.31, 2.02)	0.82
No breeding of chicken	33.4% (79/236)			3.8% (9/236)			19.7% (45/236)		
Possession of a freezer	25.3% (25/99)	0.63 (0.36, 1.09)	0.11	2.0% (2/99)	0.43 (0.09, 2.07)	0.34	33.3% (25/99)	4.36 (2.29, 8.29)	<0.0001
No possession of a freezer	34.9% (61/175)			4.6% (8/175)			10.3% (18/175)		
Electricity at home	32.9% (86/269)	5.19 (0.28, 94.90)	0.33	3.7% (10/269)	0.45 (0.02, 8.60)	1.00	19.0% (51/269)	2.59 (0.14, 47.67)	0.59
No electricity at home (-)	0.0% (0/5)			0.0% (0/5)			0.0% (0/5)		
Delivery via Caesarian section	19.0% (4/21)	0.50 (0.16, 1.54)	0.33	0.0% (0/21)	0.58 (0.03, 10.26)	1.00	61.9% (13/21)	9.39 (3.63, 24.26)	<0.0001
Vaginal delivery	32.0% (78/244)			3.7% (9/244)			14.8% (36/244)		
Country: Ivory Coast	33.6% (75/223)	1.84 (0.89, 3.80)	0.13	4.0% (9/223)	2.10 (0.26, 16.99)	0.69	10.3% (23/223)	0.094 (0.047, 0.190)	<0.0001
Country: Ghana	21.6% (11/51)			2.0% (1/51)			54.9% (28/51)		

95%-CI = 95%-confidence interval. % = percentage. N = number.

**Table A7:** Association of nondichotomous nominally scaled parameters with recorded positivity for bacterial resistance determinants, sexually transmitted infections and schistosomiasis (calculated applying the Chi-square test of independence).

	Bacterial Resistance Determinants			Sexually Transmitted Infections			Schistosomiasis		
	Proportion of Samples Positive for Bacterial Resistance Determinants in % (n/n)	Chi-Square Value	Significance P	Proportion of Samples Positive for Sexually Transmitted Infections in % (n/n)	Chi-Square Value	Significance P	Proportion of Samples Positive for Schistosomiasis in % (n/n)	Chi-Square Value	Significance P
Floor at home: earth/sand	26.7% (4/15)	0.24	0.89	0.0% (0/15)	1.21	0.55	2.0% (3/15)	4.56	0.10
Floor at home: vinyl/tiles	30.0% (15/50)			2.0% (1/50)			8.0% (4/50)		
Floor at home: cement	32.1% (67/209)			4.3%% (9/209)			21.1% (44/209)		
Toilet: pit latrine	36.5% (54/148)	4.21	0.12	5.4% (8/148)	3.48	0.18	12.8% (19/148)	7.15	0.03
Toilet: improved pit latrine	27.4% (20/73)			2.7% (2/73)			24.7% (18/73)		
Toilet: flush toilet	22.6% (12/53)			0% (0/53)			26.4% (12/53)		
Water source: tanker	0.0% (0/1)	2.74	0.43	0.0% (0/1)	0.93	0.82	0.0% (0/1)	7.09	0.07
Water source: well	22.2% (2/9)			0.0% (0/9)			44.4% (4/9)		
Water source: piped water	32.2% (84/260)			3.8% (10/260)			17.3% (45/260)		
Water source: other (also not surface water or bore hole)	0.0% (0/4)			0.0% (0/4)			50.0% (2/4)		
Occupation: housewife	31.0% (18/58)	4.24	0.38	3.4% (2/58)	0.10	1.00	13.8% (8/58)	3.64	0.46
Occupation: farmer	100% (1/1)			0.0% (0/1)			0.0% (0/1)		
Occupation: trader	32.2% (19/59)			3.4% (2/59			13.6% (8/59)		
Occupation: salary worker	20.0% (6/30)			3.3% (1/30)			23.3% (7/30)		
Occupation: other	33.3% (42/126)			4.0% (5/126)			22.2% (28/126)		
Education: none	36.7% (40/109)	3.60	0.31	2.8% (3/109)	2.59	0.46	15.6% (17/109)	2.43	0.49
Education: primary	25.9% (21/81)			3.7% (3/81)			23.5% (19/81)		
Education: secondary	32.8% (20/61)			6.6% (4/61)			19.7% (12/61)		
Education: tertiary	21.7% (5/23)			0.0% (0/23)			13.0% (3/23)		

% = percentage. n = number.
